# Benthic communities on restored coral reefs confer equivalent aesthetic value to healthy reefs

**DOI:** 10.1038/s41598-025-06373-3

**Published:** 2025-07-01

**Authors:** Cut Aja Gita Alisa, Tries B. Razak, Nicolas Mouquet, Nicholas A. J. Graham, Christopher R. Hemingson, David Mouillot, Mars Coral Restoration Project monitoring team, Lily Damayanti, Mochyudho E. Prasetya, Permas B. Maulana, Arfan Hamka, Angga Dwiyanto, Andi T. Abeng, Irwan, R. Madjid, Esya A. Hidayat, Hardin Lakota, Cicilia V. Parrangan, Andi M.A. Pratama, Hamzah, Suandar, Beginer Subhan, Neviaty P. Zamani, Rindah Talitha Vida, Timothy A. C. Lamont

**Affiliations:** 1 Department of Marine Science and Technology, Faculty of Fisheries and Marine Science, IPB University, Bogor, Indonesia; 2 Indo Ocean Foundation, Desa Toyapakeh, Nusa Penida, Klungkung, Bali, Indonesia; 3 General Organization for Conservation of Coral Reefs and Turtles in the Red Sea, Jeddah, Saudi Arabia; 4 MARBEC, Univ Montpellier, CNRS, Ifremer, IRD, Montpellier, France; 5 CESAB – FRB, Montpellier, France; 6 Lancaster Environment Centre, Lancaster University, Lancaster, UK; 7 Department of Marine Science, Marine Science Institute, the University of Texas at Austin, Port Aransas, TX USA; 8 Maritime and Marine Science Center of Excellence of Pattimura University, Ambon, Indonesia; 9 Mars Sustainable Solutions, Makassar, Indonesia

**Keywords:** Aesthetic value, Colours, Coral reef, Deep learning, Restoration, Marine biology, Ecosystem services, Restoration ecology

## Abstract

Coral reefs are valuable ecosystems that provide diverse ecosystem services to people. For example, many reefs have exceptionally high tourism value, attracting visitors to experience their ecologically and visually rich reef habitat. However, human-induced degradation can alter ecosystem services, such as when damaged reefs lose their visual appeal. Coral restoration has become a common response to reef degradation, but restoration success is usually evaluated based on coral cover increases rather than ecosystem service recovery. Here, we quantify the aesthetic value of restored reefs at one of the world’s largest coral restoration projects, compared to nearby healthy and degraded reefs. Using deep learning models trained on people’s visual preferences, we estimated the aesthetic value of coral reef benthic photographs with high prediction accuracy (R^2^ = 0.95). Restored reefs exhibited aesthetic value that was statistically equivalent to healthy reefs and significantly higher than degraded reefs. High aesthetic value was primarily driven by colour diversity and live coral cover, which were both higher in healthy and restored reefs than degraded reefs. Taken together, these results demonstrate the recovery of aesthetic value towards a healthy state after large-scale restoration, indicating that coral restoration can support vital tourism services and well-being contributions to people.

## Introduction

Coral reefs are among the most diverse and valuable ecosystems in the world^1,2^, providing benefits to roughly one billion people^2–4^ through food, coastline protection from wave exposure, tourism, and recreational and cultural heritage^5^. Recreation and tourism revenue provided by coral reefs are estimated at US$36 billion per year^6^. This high tourism potential relies in a large part on the aesthetic value of coral reefs^7,8^. Every year, millions of domestic and international tourists visit coral reefs worldwide to experience their diverse marine life.

Yet, many coral reefs around the world are threatened or already severely degraded^9,10^. Reefs worldwide are damaged by marine heatwaves, increasingly severe tropical storms, destructive fishing, poor water quality, and outbreaks of disease and coral predators^5,11–13^. As coral reefs around the world degrade, the ecosystem services they provide also change^2,14^. For example, there is a risk that degraded reefs have reduced aesthetic value^15^, potentially discouraging tourists from visiting and declining income from the tourism sector. Reefs with altered aesthetic value are also likely to have altered cultural value to people^7^.

Coral reefs that have been damaged can often recover from degradation events if the environmental conditions are favourable and the extent of the disturbance is not severe^16–19^. However, when disturbance is severe, reefs can transition to alternative stable states, or ecosystems dominated by turf algae, macroalgae or other non-coral organisms^20,21^. One dominant and persistent degraded state is characterised by a high proportion of loose rubble that prevents the survival of coral recruits and thus reef recovery^22,23^. Ecological phase shifts of this nature can lead to loss of coral cover and changes in reef structure^24^, with knock-on impacts for other reef organisms, such as fish, that rely on corals for shelter and feeding^25^.

In response to the multiple threats impacting coral reefs worldwide, scientists and conservationists are considering new ways to manage these ecosystems through different conservation and intervention strategies^26,27^. This includes a range of physical interventions that aim to increase coral cover where natural recovery does not occur, collectively known as coral reef restoration^26,28,29^. Indonesia has more coral reef restoration programmes than any other country in the world, and these projects use a suite of different restoration methods^30^. One of the largest programmes in the country has achieved extensive increases in coral cover by using modular metal frames called “Reef Stars” to stabilise loose rubble and plant fragments of live corals^31^; this method has now been replicated in many locations across Indonesia^30^.

Many coral restoration projects quantify success exclusively through measurements of coral cover, coral growth, or species diversity, which do not describe the full range of coral reef functions and contributions to people at a broader scale^32^. Although a few studies are starting to quantify wider ecosystem functions, such as carbonate production and habitat provision^33,34^, these remain in the minority. While tourism and cultural values are well established as important motivators for coral restoration, no study to date has quantified the recovery of aesthetic value when reefs are restored. Therefore, this study uses a new method to quantitatively assess an important ecosystem service provided by restored coral reefs.

Aesthetic value in natural ecosystems can be assessed using both quantitative and qualitative approaches^8,35^. Qualitative methods, such as expert evaluations and open-ended surveys, allow researchers to explore how individual perceptions vary based on socio-demographic traits^36,37^. However, such qualitative methods have limitations in generalisation and reproducibility^38^. Other approaches incorporate broader perspectives of a wider range of people through online surveys and questionnaires, which assess aesthetic judgments on standardised photographs of benthic habitat, fishes, or seascapes^8,35,39–41^. Further, emerging approaches rely on computational metrics to predict aesthetic value based on features and characteristics of photographs; such as colour intensity and diversity, relative size, and fractal dimension of the image subject^42^. Deep learning algorithms have also been used to create a predictive model of aesthetic value^38,43^.

In this study, we compared the aesthetic value of benthic community photographs in three conditions: healthy, degraded, and restored reefs. We also identified visual features of photographs relating to colour and shape that have strong power to predict aesthetic value. Specifically, this study addresses three key topics: 1) Quantifying the recovery of aesthetic value of benthic communities in restored coral reef habitats. 2) Identifying the key factors influencing the aesthetic value of benthic communities. 3) Providing a better understanding of the extent to which coral reef restoration can recover some of the perceived aesthetic value of healthy reef ecosystems. Taken together, this study facilitates a better understanding of how coral reef restoration can help to retain and recover a reef’s visual appeal, as a culturally significant and economically valuable contribution to people^44,45^.

## Materials and methods

### Study site

**Fig. 1:**
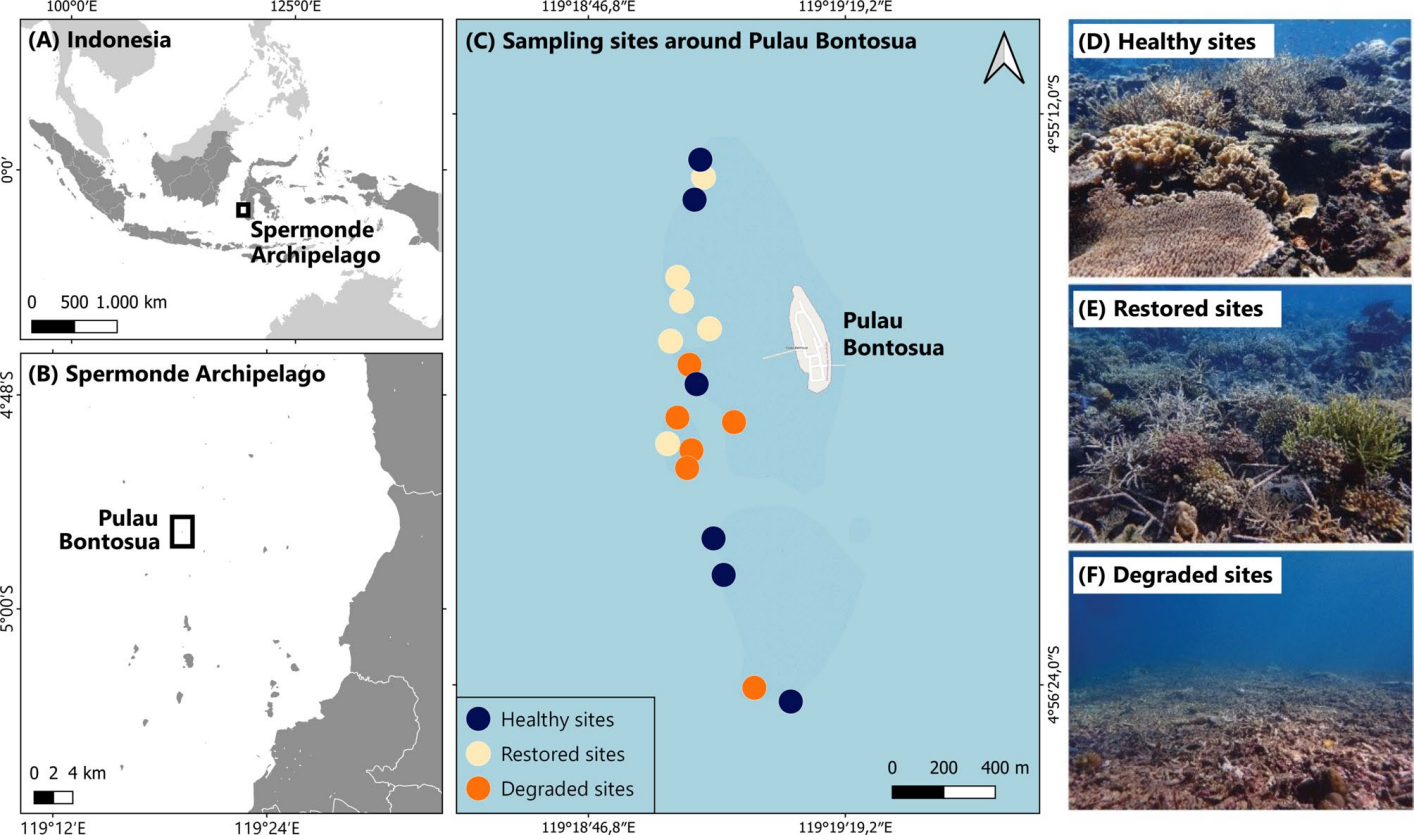
Map of the study system and its location within Indonesia. Shown are Indonesia (**a**); the location of Pulau Bontosua within the Spermonde Archipelago (**b**); the individual sampling sites around Pulau Bontosua (**c**), and example of reefs conditions of healthy (**d**); restored (**e**); and degraded (**f**) reefs at the site. Note the Reef Star structures in the foreground picture in (**e**).

This study was based at one of the world’s largest coral reef restoration programmes – the Mars Coral Reef Restoration Project at Pulau Bontosua (Bontosua Island), part of the Spermonde Archipelago in Central Indonesia (Fig. 1). The reefs around Pulau Bontosua comprise a patchy matrix of three different habitat types: healthy reefs with extensive coral cover and no recent history of damage; degraded reefs, dominated by loose rubble due to a history of extensive blast fishing and coral mining; and previously-degraded reefs that have been subject to active coral restoration for several years. The restoration used at these sites is the result of a community-led programme that has been using the Mars Assisted Reef Restoration System (MARRS)^46,47^. This method is designed to rapidly re-build coral cover at large scales on degraded rubble fields^31^, through a highly maintained and monitored program^46^. The method uses modular hexagonal metal frames, known as ‘Reef Stars’, which are interlinked to stabilise loose rubble, and provide an attachment point for coral fragments. This approach has led to a significant increase in coral cover across locations at this study site spanning 7,000 square meters, with documented increases in coral cover from approximately 10% to 60%^31^.

We chose 18 sampling sites on the reefs within this study system around Pulau Bontosua. The sites were all located within a small spatial reef area of 0.79 km^2^ (darker blue area in Fig. 1C), meaning that the water quality and weather on any given sampling day was consistent across all sites. These sampling sites comprised three habitat conditions: 6 sites of natural healthy coral reefs with extensive coral cover and no recent history of blast fishing (mean ± SD coral cover: 70.46% ± 23.02%; mean ± SD rubble cover: 5.89% ± 14.70%); 6 sites of degraded reefs, characterised by extensive fields of loose rubble caused by heavy blast fishing and coral mining in recent history (mean ± SD coral cover: 11.71% ± 21.27%; mean ± SD rubble cover: 74.91% ± 29.64%), and 6 sites of restored reefs where restoration had been carried out for 3–4 years prior to this study, using the MARRS approach (mean ± SD coral cover: 61.32% ± 22.01%; mean ± SD rubble cover: 10.36% ± 15.92%). For full details of the history of these sites and the restoration approach used, see^46^. For descriptions of the impact of restoration on other aspects of reef ecology at this site, see^31^ for recovery of coral cover and composition^33^, for recovery of carbonate production^34^, for recovery of structural complexity, and^48^ for recovery of reef soundscapes.

### Reef surveys

At each sampling site, a standardised protocol was used to take photographs of the benthic habitat. A colour standard slate (The Grey White Balance Colour Card 24; https://www.greywhitebalancecolourcard.co.uk/) was affixed to a 50 cm^2^ PVC quadrat and placed onto the substrate. This colour standard slate contains 24 different colour patches encompassing most of the visible light spectrum, including white and black. At each site, we took 50 photographs of the substrate, positioned every metre along a 50 m long transect line that ran perpendicular to the reef crest, resulting in 900 photographs. The quadrat was photographed from directly above using a waterproof digital camera (Olympus TG-5) to capture the entire quadrat and the colour slate in a planar view. We used the camera’s underwater-auto settings, which automatically adjust white balance, colour correction, and exposure to compensate for the light absorption and scattering effects of water. Since all sites were relatively shallow, with depths ranging from 2 to 4 m (for specific details on depth and photograph dates for each site, see Table S1 in Supplementary Information), the blue light-filtering effect of water did not have a notable effect^49^. An ANOVA test showed no significant differences in the depths of sites across different habitat categories (F(1, 879) = 0.035, p > 0.05). Weather conditions during the sampling period were consistent – calm weather with clear skies and no rain – ensuring that the photography conditions were the same across all habitat types.

In pilot trials, we tested several of the camera’s preset shooting modes and settings, and found that the preset “underwater-auto” setting was best suited to capturing the natural colours of the corals. This underwater-auto setting applies an automatic colour adjustment process during the capture of the photograph, that in shallow-water conditions accurately replicates the colours experienced by the human eye in these environments. The presence of a colour standard slate increases the effectiveness of automatic colour balancing algorithms of this nature. As such, this setting generated photographs that are a realistic representation of how human observers would view this environment in real life. To confirm that the underwater-auto colour adjustment process was working as expected in each photograph, the same surveyor (C.A.G.A.) carried out visual quality control checks by comparing the appearance of the colour standard slate in each photograph to its appearance in situ when underwater. If the colour standard slate in the photograph appeared noticeably different to how it looked underwater, we judged that the underwater-auto settings had failed in that instance, and discarded that photograph from further analysis. This process led to the discarding of 17 of the 900 photographs, resulting in 883 photographs across all sites. In this way, our photo standardisation process took advantage of both standardised calibration algorithms (the universal application of the same colour-adjustment setting in the camera) and human-centred decision-making process (the quality control checks). As such, we could be confident that the photographs used in the survey were meaningful representations of how this environment would be perceived by human observers in real-world conditions.

After the quality control checks, each photograph was cropped to contain only the 50 × 50 cm area within the quadrat. Following this, we equalized the resolution of the photographs to be 700 × 700 pixels at 92 dpi, ensuring that all the photographs contained the same number of pixels.

### Questionnaires

We evaluated the aesthetic value of each photograph of benthic habitat, through an anonymous online questionnaire to the general public. The online questionnaire was pre-tested between 14 April to 14 May 2023 and open to the public for responses from May 15^th^ to September 30^th^ 2023. The survey was available in English and French on a dedicated website (https://www.biodiful.org/#/survey/15/present), which had been previously established and used in earlier studies^35,39,43^. English was chosen to reach a broad international audience, while French was included due to an existing network of participants from previous studies using the same platform. All methods were carried out in accordance with the Economic and Social Research Council’s Research Ethics Guidelines, available at www.ukri.org/councils/esrc/guidance-for-applicants/research-ethics-guidance/. The Department of Marine Science and Technology at IPB University approved the experimental protocols. All participants were informed about their participation in this research and consented to the use of their anonymised answers. Informed consent was obtained from all subjects prior to their participation in the survey.

This survey comprised two sections. The first section asked participants to compare the aesthetic value of different photographs; participants were presented with a randomly-sampled pair of photographs from a subset of 300 of the standardised benthic habitat photographs (100 from each habitat category: natural healthy reefs, restored reefs, and degraded reefs). These photographs were selected randomly from the total pool of 883 photographs. The same subset of 300 photographs was consistently used for all participants in the survey. In each pair, participants were asked to choose the photograph that they found most beautiful. This exercise was repeated 30 times, so each participant indicated an aesthetic preference between 30 random pairs of photographs. For an example screenshot of the presentation of these paired photographs, see Fig. S1-S3 in Supplementary Information.

The second section consisted of questionnaires designed to collect information on the sociocultural background and relevant experience of participants, aiming to examine whether these factors had any influence on aesthetic preferences. This included asking participants their gender, age, country of residence, education level, diving experience, and self-declared knowledge about corals (for a full list of questions and possible responses, see Supplementary Information). Participants were asked if they have problems with colour perception (colour vision deficiency). Responses from individuals who reported such difficulties were excluded from the analysis, resulting in the removal of 96 out of 3,348 responses.

Our survey was disseminated extensively via several different platforms. Firstly, the survey was sent via email to the authors’ contacts and various mailing lists in multiple different countries (including scientific societies, universities, NGOs, and special interest groups). The survey was also shared widely across social media platforms (Instagram, Twitter, LinkedIn, Facebook and Strava). We chose social media for rapid dissemination of information across a global audience. However, we acknowledge that this approach may introduce bias by attracting those who are more active online. Participants’ choices were not visible to other participants in the survey, meaning that there could be no influence of others’ responses on any participants’ answers. Participants were asked not to carry out the survey more than once but were encouraged to further distribute it among their family and friends after completing it. This encouragement to share widely precluded the possibility of tracking exactly where the survey had been shared; however, for a non-exhaustive list of example platforms on which it was shared, see Table S2 in Supplementary Information. The survey was completed by 3,348 respondents from 107 different countries, with the majority in France (28.5%), Indonesia (16.2%), UK (14%), USA (9.9%) and Australia (7.1%). Respondents represented a wide spread of different levels of education: secondary school (4.81%), high school (10.39%), bachelor’s degree (27.72%), master’s degree (32.35%), PhD (24.73%); knowledge of corals: poor (12.66%), low (25.06%), average (26.28%), good (23.18%), and excellent (12.81%), as well as diving experience: diving (53.32%), only snorkelling (24.37%), or none (22.31%) (See Fig. S5 in Supplementary Information). For a full breakdown of participant numbers by each sociocultural variable, see Table S3 in Supplementary Information.

### The rating of aesthetic value

The results of the online survey (aesthetic preferences between pairs of photographs) were used with an Elo Algorithm^50^ to evaluate and rank photographs, using the EloChoice v0.29.4 R package^51^ with 1,000 bootstrappings. The Elo Algorithm is a rating system primarily used to create rankings based on pairwise comparisons of individuals, commonly used for measuring chess players’ performances. This Elo rating (referred to as the ‘aesthetic rating’ hereafter) is a numerical system in which differences in ratings may be converted into scoring or winning probabilities^50^.

Following Langlois et al., (2021)^38^, the 300 photographs that were evaluated in the online survey and their computed aesthetic ratings were used as a training dataset for a deep learning algorithm that predicted the aesthetic ratings of the whole dataset of 883 photographs. Deep learning algorithms are well-suited for tasks related to identification and classification of objects, and prediction of continuous variables^52^ and are widely used across a range of research domains, including ecology^53^. In our study, we used a Convolutional Neural Network (CNN) deep learning algorithm, which are generally effective for computer vision tasks such as recognizing objects, patterns, and features in images^52–54^. We applied transfer learning by fine-tuning a CNN pre-trained on ImageNet^55^, allowing the model to leverage existing knowledge and adapt to our task of predicting the aesthetic value of coral reef photographs. We selected the Residual Networks (ResNet) architecture because of its high accuracy and efficiency in image recognition tasks^56^.

Our dataset was partitioned as 70% into training (210 photographs), 15% into validation (45 photographs), and 15% into testing (45 photographs). Given the low number of images in our training dataset, we used data augmentation techniques from the ‘torchvision.transforms’ library in Python to generate diverse variations of each image, specifically RandomRotation, RandomHorizontalFlip, and RandomVerticalFlip. This effectively increased the diversity of the training data, allowing the algorithm to learn more features. This technique is also commonly used in similar studies with small datasets^38^. Data augmentation, fine-tuning of the model, and prediction of the aesthetic values were carried out using Python 3.7, Pytorch 1.4.0, and torchvision 0.5.0 using the same procedure as in^38^. We used the r^2^ of the linear regression between the values predicted by the model and the values of the testing set (on which the model was not trained) to estimate the final accuracy of the model.

### Photograph feature extraction

Several visual features of photographs that potentially linked to predicted aesthetic rating^39,41,42,57,58^ were calculated. These features included those related to colour, shape, or both colour and shape. Initially, we calculated values for nine photograph features: (1) Simpson diversity of colours, (2) percentage of black, (3) percentage of blue, (4) percentage of green, (5) percentage of yellow, (6) percentage of grey, (7) count of colours, (8) count of coral morphology, and (9) percentage of live hard coral.

To calculate colour features (numbered as 1–7 in previous sentence), we adopted an approach to assess ‘community colouration’, based on the methods used by Hemingson et al., (2021)^15^. We quantified the colouration of the benthic community within each quadrat by measuring the proportion of different colours present in each photograph. To accomplish this, we used the *getHistList* function from the *colordistance v 1.1.2* R package^59^, which measures the number of pixels within specific regions of the red, green, blue (RGB) colourspace. This approach provides an objective and comparative tool for analysing colour distributions^15^.

This process generates a ‘colour thumbprint’ for each photograph by recording the relative amounts of each colour present^15^. Each colour thumbprint comprises 64 bins that each represent a different section of the RGB colour space, as detailed in Table S5. Several of these bins were then combined to form distinct colour groups: black, blue, yellow, green, and grey. The proportion of the black colour group was indicative of the amount of shadow in each photograph, so we used this as a proxy for structural complexity; benthic features with a high structural complexity cast a lot of shadow, creating a lot of black pixels in photographs. The proportion of the grey colour group was indicative of the rubble or sand coverage, as these were the dominant benthic features in the grey colour space. The blue, yellow, and green colour groups were indicative of the colours of common corals in this ecosystem; each of these three groups accounted for more than 1% of the pixels in photographs overall. For full details of colour groupings, see Table S6 in Supplementary Information.

We also calculated the Simpson diversity index based on colour proportions in each photograph. The Simpson diversity index measures the probability that two randomly selected individuals from a sample will belong to the same species^60^. In this study, the Simpson diversity index was applied to evaluate colour diversity by quantifying how evenly different colours were distributed across habitats. We also calculated ‘count of colours’ to measure how multi-coloured each photograph was; for this metric, we counted the number of colour bins that contained more than 1% of the total pixels. Including both the Simpson diversity index and the count of colours highlights different aspects of the colour diversity of a photograph. The Simpson diversity index indicates whether the aesthetic value comes from a balanced mix of colours or is dominated by a few colours, while the count of colours provides a simple measure of richness, irrespective of how much of the photograph is taken up by each colour.

Features related to coral morphology were calculated by several approaches. The feature ‘percent of image in shadow (proxy for structural complexity)’ was determined from the proportion of the black colour group, as black areas in the photographs represent shadows cast by coral structures. We also counted the number of different morphologies present in each photograph, by quantifying the presence or absence of branching, digitate, encrusting, foliose, massive, submassive, mushroom and tabulate coral morphologies. Further, we quantified the percentage cover of live hard coral using CoralNet software (coralnet.ucsd.edu). We randomly placed 50 points on each photograph and recorded the benthic cover beneath each point, and then doubled this value to convert into percentage cover values. Benthic cover was categorized into types of living biota (hard coral, soft coral, sponge, crustose coralline algae, gorgonian), abiotic components (dead coral, rubble, sand, rock), or other abiotic components such as metal frames. The percentage of live hard coral was the only metric considered in the regression analysis for photograph feature extraction, because the coverage of all other benthic categories was comparatively very low (average 0.4% or lower).

### Statistical analysis

To test the effect of socio-cultural background on the online survey participants’ choices, we computed a generalized linear mixed model (GLMM) with a binomial error structure in which the photograph was considered as a random effect variable to order the socio-cultural variables according to their individual effect on the response variable (*glmer* function from the *lme4 v 1.1–26* R package). To reduce the number of combinations tested, we created age categories (< 12; 13–18; 19–39; 40–59; > 60 years) and country categories (France, Indonesia, UK, USA, Australia as individual categories which together represented 75% of the respondents, and an ‘Other countries’ category). The final socio-cultural variables used were: gender (categorial), age class (ordered), education level (ordered), diving experience (categorial), country (categorial) and knowledge about corals (ordered). This analysis of variance for the GLMM model (the function *Anova* from the *car v3.0–9* R package) showed no effect of any of the socio-cultural variables, nor any two-way interactions among them (Table S4 in Supplementary Information). This allowed us to pool all the respondents answers together to compute the aesthetic ratings.

To assess the individual effect and ranking of features (9 features) in explaining the variance in predicted aesthetic ratings for the 883 photographs, we used a multiple regression approach. Initially, we conducted a backward stepwise correlation matrix using Pearson correlation coefficients among all features. When two features were correlated higher than 0.7 or lower than -0.7 as a threshold, we then individually tested the correlation between those features and the predicted aesthetic rating. We kept the feature with a higher correlation to aesthetic value, and eliminated the feature with the lower correlation. We repeated this iteration of removing correlated features until none of the remaining features correlated with each other at a level that exceeded the threshold (0.7).

The stepwise deletion resulted in the ‘Percentage of grey’ feature being removed due to its strong inverse correlation with the ‘Simpson diversity of colours’ feature. The remaining eight features were not correlated above the 0.7 threshold level, so were included in further analyses: (1) Simpson diversity of colours, (2) percentage of black, (3) percentage of blue, (4) percentage of green, (5) percentage of yellow, (6) count of colours, (7) count of coral morphology, (8) percentage of live hard coral.

Next, we conducted a backward stepwise linear regression model (with a Gaussian response) to explain the impact of these eight variables on the predicted aesthetic rating and derive a minimal adequate model. Independent variables (eight features) were scaled, while the dependent variable (predicted aesthetic rating) was kept in its original, unscaled form. Through an iterative process, non-significant features (p > 0.05) were eliminated, resulting in a final model that retained the features that were most closely linked to the predicted aesthetic rating. See Table S7 in Supplementary Information for the full photograph feature extraction models. The remaining 6 features were: (1) Simpson diversity of colours, (2) percentage of black, (3) percentage of blue, (4) count of colours, (5) count of coral morphology, (6) percentage of live hard coral.

Each of the visual features (Simpson diversity of colours, percentage of black, percentage of blue, count of colours, count of coral morphology, percentage of live hard coral) was compared across the healthy, degraded, and restored sites using mixed-effect models. If data were normally distributed, linear mixed effects models (LMM) were used, using *lmer* function from the *lme4 v 1.1.35.1* R package. If data were positively skewed, Poisson or Gamma distributed generalised linear mixed-effects models (GLMM) were used, using *glmer* function from the *lme4 v 1.1.35.1* R package. Visual examination of histograms and normal quantile plots of model residuals was used to confirm model goodness-of-fit. In each model, habitat type (healthy, degraded, or restored) was included as a fixed effect, while site ID was included as categorical random effect. The LMM model was defined as:

$$Response \, variable \, \sim \, fixed \, effect \, + \, \left( {\left. 1 \right| \, random \, effect} \right)$$

applied to: 1) Aesthetic rating: aesthetic rating ~ habitat + ( 1 | site ID), 2) The percentage of black: the percentage of black ~ habitat + ( 1 | site ID), 3) The percentage of live hard coral cover: The percentage of live hard coral cover ~ habitat + ( 1 | site ID). The GLMM model was defined as:

$$Response \, variable \, \sim \, fixed \, effect \, + \, \left( {\left. 1 \right| \, random \, effect} \right), \, family \, = \, x$$

applied to: 1) Simpson diversity: Simpson diversity ~ habitat + (1| site ID), family = Gamma(link- “log”), 2) The percentage of blue colour: The percentage of blue colour ~ habitat + (1| site ID), family = Gamma(link- “log”), 3) Number of colour categories: Number of colour categories ~ habitat + (1| site ID), family = Poisson, 4) Number of coral morphologies: Number of coral morphologies ~ habitat + (1| site ID), family = Poisson.

The overall effect of habitat type on the dependent variable was tested using ANOVA comparisons to null models that were identical except for the omission of the fixed term. If this comparison was statistically significant (*p* < 0.05), post-hoc Tukey’s HSD testing followed to provide between-habitat comparisons.

## Results

According to the online survey results, there was no significant effect of age, gender, nationality, education level, or familiarity with coral reefs when selecting the most visually appealing photograph from a given pair (see Table S4 in Supplementary Information for full model). For all remaining analyses we therefore grouped aesthetic ratings from all respondents.

**Fig. 2:**
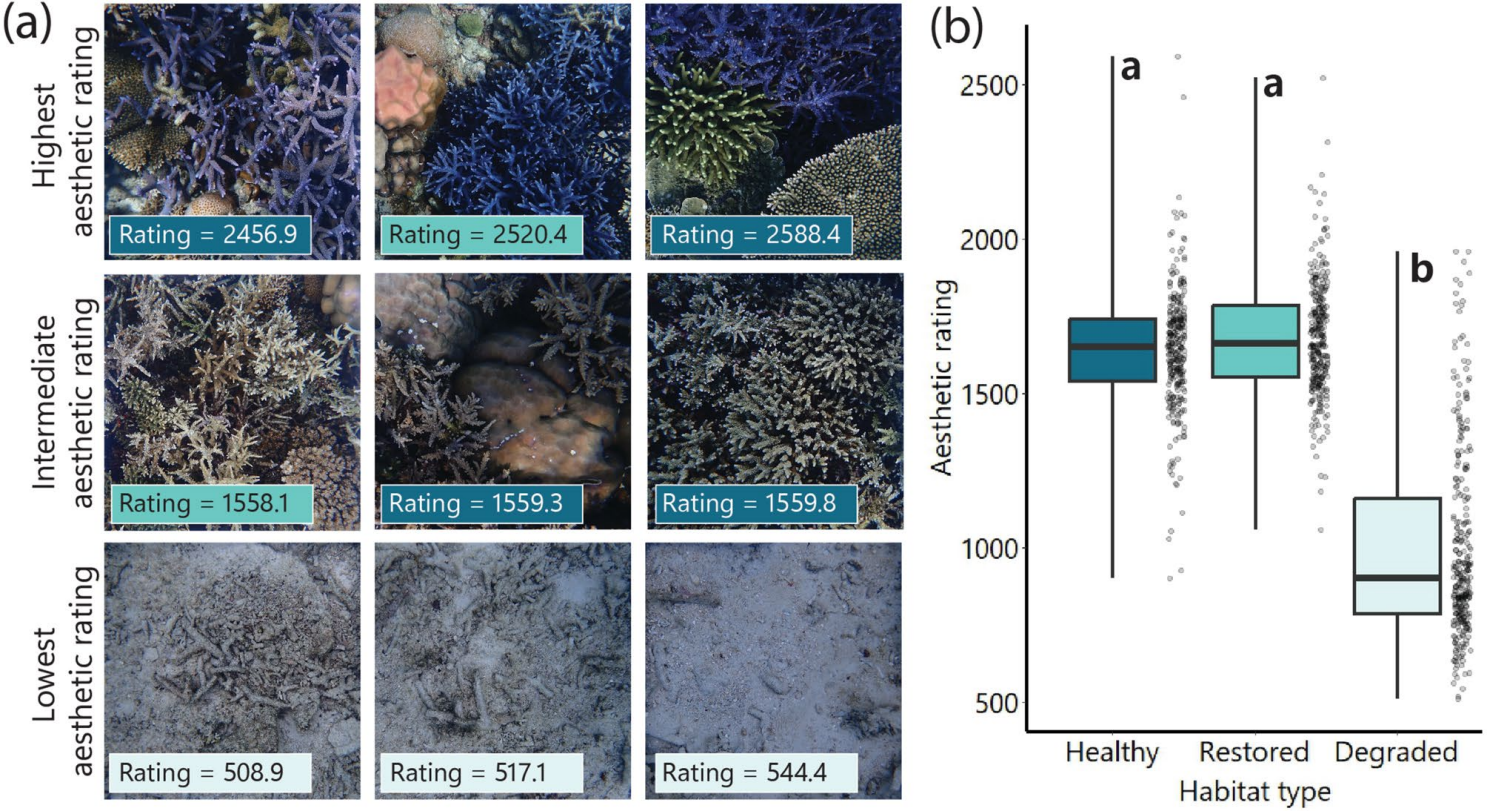
Aesthetic rating of benthic habitat. (**a**) Examples of photographs with varying predicted aesthetic ratings, spanning from 508.9 (lowest aesthetic rating) to 2588.4 (highest aesthetic rating). The top row are the photographs with the three highest predicted aesthetic ratings; the second row are photographs with intermediate predicted aesthetic ratings; and the third row are the photographs with the three lowest predicted aesthetic ratings. In each photograph, the aesthetic rating is given in the corner, with the background colour indicating whether the photograph is from a healthy (dark blue), restored (light blue), or degraded (grey) habitat. (**b**) Predicted aesthetic ratings of photographs from healthy, restored, and degraded reefs. Each point represents the modelled aesthetic rating of one photograph, and boxplots represent the median (central line), interquartile range (boxes) and full range (whiskers) of the data. A linear mixed model revealed a significant effect of habitat type on aesthetic rating (p < 0.001); different letters represent significant differences in Tukey’s HSD post-hoc testing. For details of full models, see Table S8 in Supplementary Information.

The predicted aesthetic ratings from the model was remarkably consistent with the actual aesthetic ratings from human scores (R^2^ = 0.95), demonstrating the method’s ability to accurately predict human-perceived aesthetic value of these photographs. The predicted aesthetic rating for all the 883 photographs ranged from 508.9 to 2588.4 (Fig. 2; Fig. S4 in Supplementary Information). The three highest predicted aesthetic ratings corresponded to photographs with high coral cover and vibrant colours, while intermediate predicted aesthetic ratings corresponded to photographs with high coral cover but fewer colours (Fig. 2A). In contrast, the three lowest predicted aesthetic ratings corresponded to photographs lacking any living coral and dominated by sand or rubble, which resulted in predominantly grey tones (Fig. 2A). Overall, there was a significant effect of habitat type on aesthetic rating (LMM: χ2 = 43.85, df = 2, p < 0.001); healthy and restored habitat had a significantly higher aesthetic rating than degraded habitat, with no significant difference between the healthy and restored habitat (Fig. 2B, full model and post-hoc comparisons in Table S8 in Supplementary Information).

**Fig. 3:**
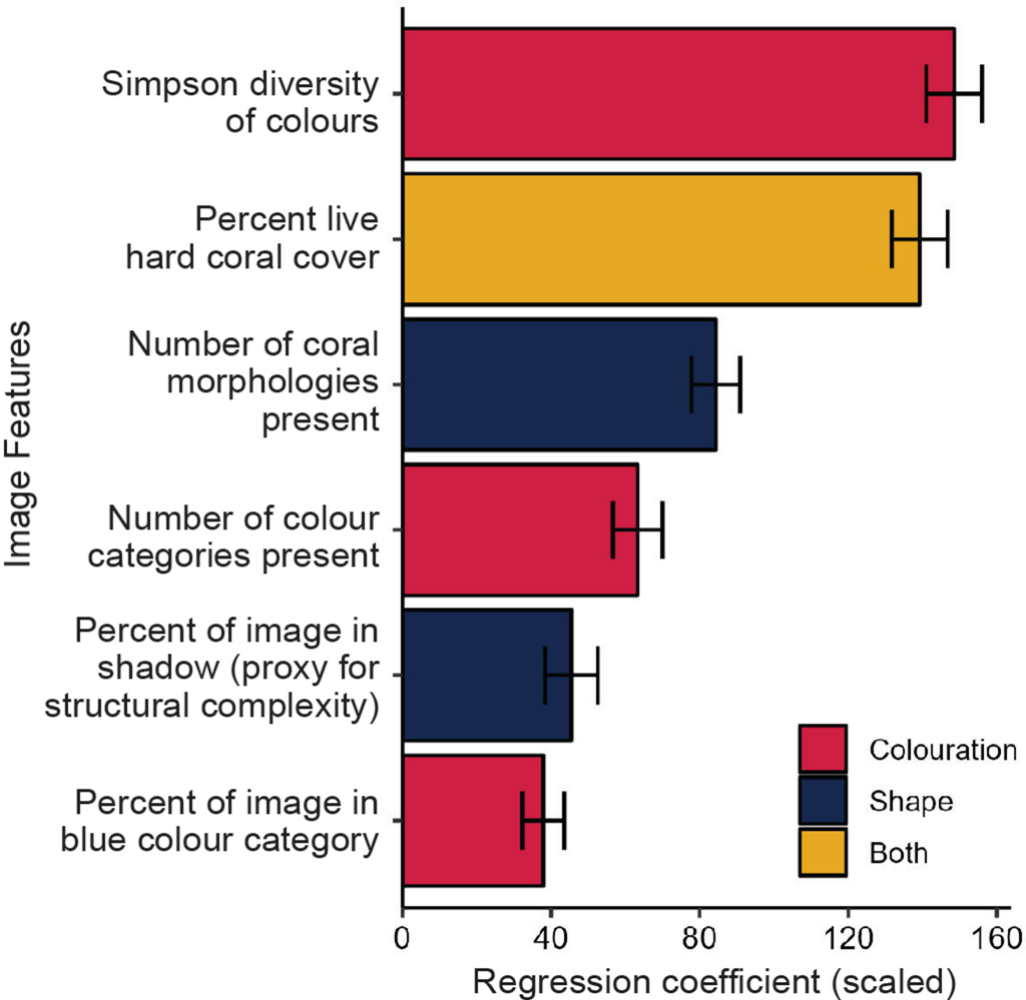
Effects of different photograph features on predicted aesthetic rating. Scaled regression coefficients (with standard errors) from a linear mixed-effects model describing the influence of six photograph features on predicted aesthetic rating. The colour of each bar indicates whether the variable is related to colouration (pink bars), shape (dark blue bars) or both (yellow bars).

After obtaining the predicted aesthetic ratings for all photographs, six significant photograph features were identified as influencing factors (Fig. 3). A linear mixed model combining all six features had strong predictive power of aesthetic rating (R^2^ = 0.84, *p-*value < 0.001, Fig. 3). Of these six features, the most influential in driving predicted aesthetic ratings were, respectively: Simpson diversity of colours, percent live hard coral cover, number of colour categories present, number of coral morphologies present, percent of the image in the blue colour category, and percent of the image in shadow (a proxy for structural complexity). The percentage of variance explained by each feature was as follows: percentage of live hard coral (30.94%), Simpson diversity of colours (28.48%), number of coral morphologies (20.70%), percentage of black (8.96%), count of colours (8.97%), and percentage of blue (1.94%).

**Fig. 4:**
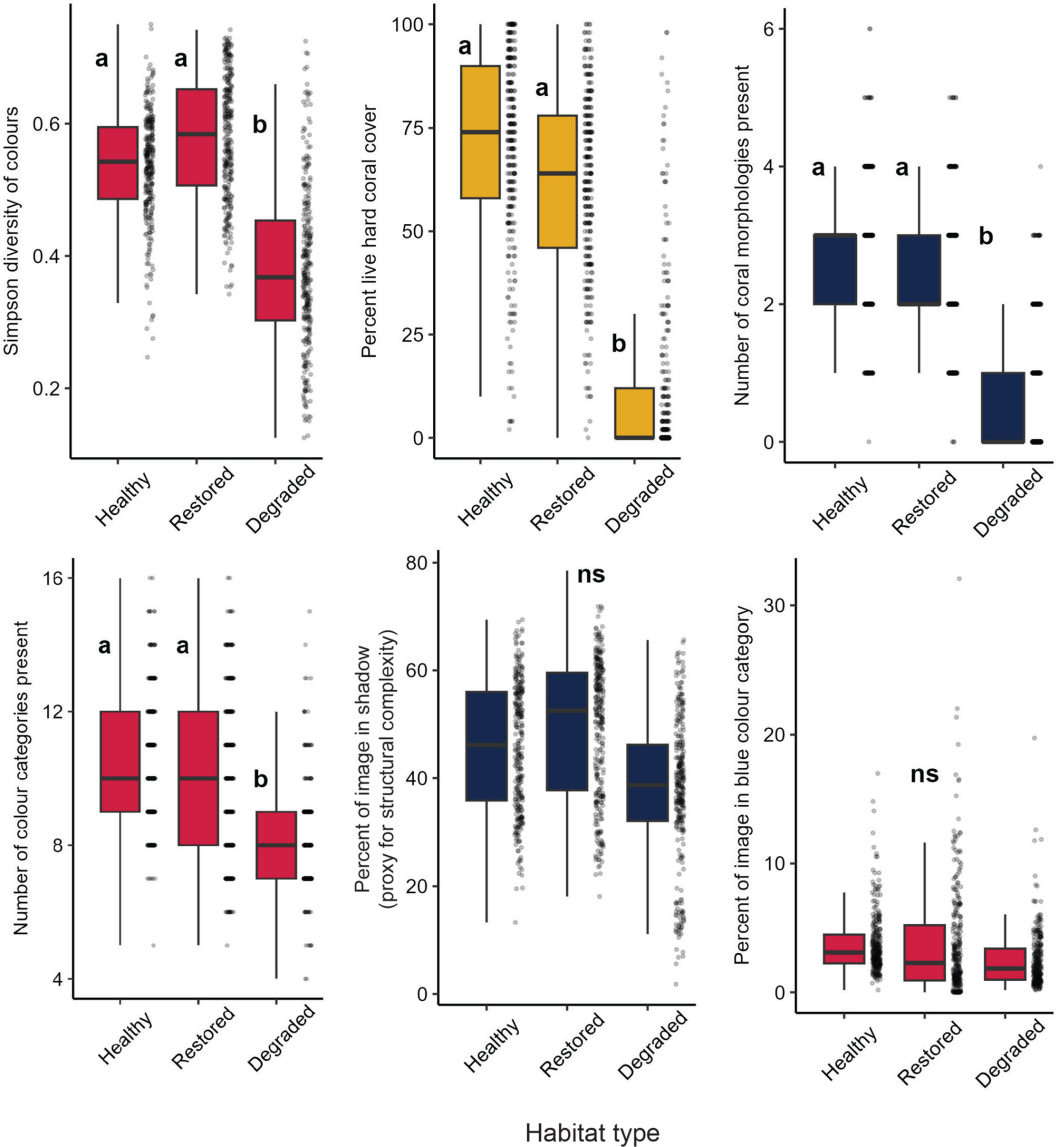
Differences in photograph visual features between habitat types. Each panel represents one of six photograph visual features that significantly impact aesthetic rating (see Fig. 3), in three different habitats. Within each panel, each point represents one photograph, and boxplots represent the median (central line), interquartile range (boxes) and full range (whiskers) of the data. Different letters represent significant differences in Tukey’s HSD post-hoc testing, following a significant effect of habitat type in mixed-effects models; ‘ns’ indicates there was no significant effect of habitat type in the model. For details of full models, see Table S8 in Supplementary Information.

Each of the six photograph features that predicted aesthetic rating were then compared amongst healthy, restored, and degraded habitats (Fig. 4). The effect of habitat on each individual variable was varied: there was a significant effect of habitat type on Simpson diversity of colours (GLMM: χ2 = 41.508, df = 3, p < 0.001), percent live hard coral cover (LMM: χ2 = 46.39, df = 2, p < 0.001), number of coral morphologies present (GLMM: χ2 = 18.033, df = 3, p < 0.001), and the number of colour categories present (GLMM: χ2 = 95.169, df = 3, p < 0.001), with post-hoc comparisons revealing in each case that healthy and restored habitats were not significantly different, but both were significantly higher than degraded habitats. However, there was no significant effect of habitat type on the percent of image in the blue colour category (GLMM: χ2 = 8.649, df = 3, p = 0.035), or the percent of image in shadow (proxy for structural complexity) (LMM: χ2 = 3.265, df = 2, p = 0.195). For full models and post-hoc comparisons, see Table S8 in Supplementary Information.

## Discussion

This study of one of the world’s largest coral restoration projects demonstrates that the aesthetic value of restored reefs’ benthic communities can be comparable to those of natural healthy coral reefs. Human-perceived aesthetic value was equivalent for healthy and restored reefs, with both of these habitats exhibiting significantly higher aesthetic rating than degraded reefs. This similarity in aesthetic ratings between healthy and restored habitats demonstrates that restoration efforts in previously degraded rubble fields can revive the visual appeal of a reef ecosystem. Furthermore, our study identifies specific visual features that are key in determining aesthetic rating, giving valuable guidance for restoration or conservation projects that aim to prioritize aesthetic value in coral reef ecosystems. We also provide a deep learning model trained on ratings from thousands of participants, that is able to predict reef aesthetic rating accurately based on photographs.

### Quantification of aesthetic value

Our findings align with a prior study where people, regardless of their knowledge or exposure to coral reef ecosystems, tended to categorize photographs of pristine and damaged coral reefs as “pleasant” and “ugly”, respectively^61^. Similarly, our online questionnaire results revealed almost ubiquitous agreement on aesthetic ratings. The fact that there was no significant difference in response between participants of different socio-cultural backgrounds suggests that coral reefs may possess a fundamental underlying aesthetic component that transcends cultures, backgrounds, or career stages. This universality of aesthetic preference has also been documented in other studies: for example, in a study on coralligeneous reefs (biogenic concretions formed through the accumulation of encrusting algae and bio-constructor animals^62^), there was no significant effect of different socio-economic backgrounds on judgements of aesthetic value^39^. In separate study, participants from diverse socio-cultural backgrounds generally agreed that the presence of landscape elements like lakes, rivers, glaciers, and natural features contributes to a higher aesthetic value in mountain ecosystems^63^. Similarly, features such as tree lines, forests, cultural buildings, and animal habitats are preferred for their aesthetic value in the landscape^64^.

The AI-driven model used in this study to predict aesthetic rating revealed a notably high accuracy score when its outputs were compared to results from the survey (R^2^ = 0.95). This cross-validated accuracy score is higher than that obtained by a previous study using the same approach on coralligenous reefs^38^. This improvement in accuracy is potentially due to the fact that this study focuses on tropical coral reefs, while the previous study focuses on temperate coralligenous reefs that show more heterogeneity in visual patterns, posing challenges for model predictions. This study also demonstrates that using convolutional neural networks effectively predicts the aesthetic value of tropical coral reefs, offering a novel tool for evaluating this important ecosystem function.

### Drivers of aesthetic value

Following our comparison of aesthetic value between different habitat types, we identified the visual features that were most influential in determining perceived aesthetic value. The diversity of colours – quantified using the Simpson diversity index – was the most important driver of predicted aesthetic ratings. Photographs with high values in this metric have a variety of different colours present in a relatively even balance, with no single colour dominating the community. Interestingly, the colour richness (the number of coral colours present in the image) was a much less influential driver than the Simpson diversity – this demonstrates that a well-balanced diversity of colour is more aesthetically pleasing than an over-dominance of one colour, even if all other colours are present in small amounts.

In addition to the importance of well-balanced colour diversity, there was also some evidence that different colour categories provoked different responses. Blue was the only colour category that exhibited a significant positive influence on aesthetic rating, unlike yellow and green which had no significant effect despite also being commonly associated with live coral. Although blue colours do increase with depth in most underwater contexts, in this study all photographs were taken at equivalent depths, and the proportion of blue was driven by the presence of blue corals rather than depth-induced colour shifts. An example of this is evident in the three photographs with highest aesthetic ratings (Fig. 2A), which all contain a noticeably high proportion of blue corals. By contrast, the grey colour category exhibited a strong negative influence on aesthetic ratings, although it was removed from the final model due to a strong inverse correlation with the Simpson diversity index (Table S7). Grey colours were often associated with non-coral benthic cover, such as rock, rubble and sand. Whilst it is possible that in other sites, non-coral benthic organisms (e.g. sponges and gorgonians) might add to the colour diversity of a reef ecosystem, this was not the case at this site. In this study, non-coral organisms comprised a small minority of the benthos (< 0.4% on average), and contributed little to colour diversity.

Following colour diversity, the second-ranking factor influencing aesthetic value was the percentage of live hard coral cover. This aligns with previous studies that have also found both coral abundance and colour diversity to be important drivers of aesthetic value^39,41,42,65^. In addition to overall coral cover, coral composition was also important in influencing the predicted aesthetic ratings. The number of different morphologies present in a given photograph – a proxy for coral diversity—was a significant predictor of visual appeal. This indicates the importance of a diversity of different coral types in determining aesthetic value. A previous study conducted on the Great Barrier Reef also highlighted the importance of diverse morphologies in driving coral aesthetics, highlighting elements such as topography and texture as important features contributing to aesthetic value^57^. In addition to the number of morphologies present, the percentage of the image in shadow (represented by the black colour category) was also a statistically significant predictor of aesthetic value. Shadows in photographs enhance visual contrast and depth perception, highlighting structurally complex reef formations with distinctive shapes, reinforcing the role of complex shapes and diverse morphologies in aesthetic appeal. Of course, in many cases there may be a degree of interdependence between all of these factors; live coral cover, colour diversity and morphological diversity are likely to positively correlate in many scenarios. However, our measures of correlation between these factors were strikingly low. For example, none of the variables in the final model had a correlation coefficient of > 0.7, and the correlation between the number of morphologies present and the number of colours present was only 0.34 (Table S7). This is likely due to instances where colour does not correlate with taxonomic diversity; for example, where multiple species of coral all share the same colour, or the same species is present in different colours^66^. As such, the weak correlations between different drivers demonstrate that no single metric is adequate to fully explain aesthetic value on its own. For example, in Fig. 2A, quadrat pictures with high coral cover and complex branching shapes receive only an intermediate aesthetic rating, possibly due to a lack of colour diversity.

### Limitations of this method

While this study is able to quantify some important aspects of coral reef aesthetic value, there are other aspects of aesthetic value that are inevitably excluded by these methods. This study focuses exclusively on benthic organisms through the lens of standardised photographs from a relatively close-up perspective (50 × 50 cm visual field). Benthic-focussed approaches like this facilitate controlled replication between habitat types, but also mean that some other factors that influence aesthetic value are not considered. For example, a previous study by Le et al., (2019)^67^, found that aesthetic value could be driven by diversity of species and form^39,57^, colour^41^, and clear water quality^57,68^. Our study includes diversity and colour, but does not consider water quality. Although we did not explicitly measure water clarity in this study, visibility on the reefs surrounding our study system (Pulau Bontosua) is consistently > 20 m due to its offshore location, situated a long way from river outflows on the main island of Sulawesi. Further, coral reefs support a wide range of taxa that inhabit the water column (rather than living on the benthos), including fishes, reptiles, mammals and invertebrates; these organisms can also serve as a key driver of the aesthetic value^69^ and cultural significance of reef ecosystems^70^. This study was not able to capture the impact of these mid-water organisms on habitat aesthetic value.

Despite these limitations in its wider scope, our benthic-focussed approach was particularly well suited to outlining differences caused by habitat restoration, because focussing on the benthos in this manner concentrates primarily on the particular aspect of reef ecology that is most altered by restoration efforts. While the effects of coral restoration on fish communities can be nuanced and difficult to quantify at small scales^71^, the effects of restoration on benthic communities are much more clear-cut and tightly coupled with restoration action. As such, limiting the scope of measurements to the benthic community ensures that we are using an optimal method for quantifying the impact of coral restoration on visual appeal. Future work incorporating additional components that influence aesthetic value, like the local fish community, water clarity, and presence or absence of marine litter or other disturbances, will add further understanding as to what practices can maximise the aesthetic value yields of restoration efforts.

It is also important to note that the context of this study site is not representative of all reef restoration projects globally. This study system involves reefs that have been restored for a greater timespan (3–4 years), and over a larger spatial area (thousands of square meters) than many other projects around the world. The project also uses a specific restoration method (Reef Stars) that is not common to all restoration projects. As such, the findings from this study may not be indicative of expected outcomes from other restoration sites that are smaller, younger, less effectively managed, or use different restoration methods. Additionally, coral reef colours and morphology may vary significantly across geographic regions; this study’s findings may not be directly applicable to other regions with different environmental conditions. However, despite uncertainty around its general application to other systems, this study exemplifies the potential benefits of long-term coral reef restoration for recovering a reef’s visual appeal, and associated benefits for cultural value and tourism opportunities.

### Implications for restoration practice

The results of this study are important for practitioners designing coral restoration efforts that have goals relating to aesthetic recovery, such as those with tourism-driven objectives. We demonstrate that when restoring reefs to maximise visual appeal, it is important to select a community of corals that will together generate a high diversity of both colour and morphology. Maximising colour and morphological diversity in this way will be particularly achievable for projects that use coral gardening methods. We suggest that where possible, practitioners collecting corals of opportunity or harvesting tissue from live donor corals should aim to collect not just a diversity of coral genera, but also a diversity of growth forms and colours within each genera. Further, when choosing outplant locations for transplanting corals from a nursery onto a natural reef, we recommend considering the colours and morphologies present in the immediate vicinity and introducing transplants that will add to this diversity. All of these approaches to maximising diversity of colour and morphology would be easy for practitioners to measure and track progress, for example by using the methods employed by this study to quantify the colours and morphologies present in standardised benthic photographs.

In some cases, approaches to maximising aesthetic value will complement approaches to maximising other ecological functions. For example, increasing the number of coral morphologies is likely to increase visual appeal while simultaneously enhancing the structural complexity of reef habitat, providing additional habitat niches for a range of reef-associated species^34,72,73^. However, colour-based approaches to maximising aesthetic value might not directly complement other measures of restoration success. For example, many corals of the same species exhibit a variety of colours, achieved through post-translational mechanisms and biochemical processes that occur in response to environmental conditions^66^. If efforts to increase colour diversity resulted in the prioritisation of coral species that have high natural variation in pigmentation (such as *Stylophora pistillata* or *Acropora tenuis*) this might not lead to an increase in species diversity in a restored reef. As such, it is important that management interventions prioritising aesthetic value must also consider other measures of restoration success, rather than assuming that a reef that looks visually appealing automatically exhibits healthy ecosystem functioning. Finally, it is also important to note that increasing tourism appeal might be a double-edged sword, because badly managed tourism could increase local stressors on the ecosystem. Tourism can have unintended negative impacts on reef ecosystems, through destructive activities such as reef walking^74^, anchor damage, and sedimentation from coastal development or construction of large tourism facilities^75^. Any development of tourism should ensure sustainable planning to avoid undermining the ecological benefits achieved by reef restoration.

## Conclusion

This study demonstrates that a large-scale, well-maintained coral restoration project can generate a benthic community that has equivalent aesthetic value to that of nearby natural healthy reefs. This aesthetic judgement is agreed upon by thousands of people from different sociocultural contexts, is inherently predictable by AI-driven algorithms, and is driven by a mixture of visual features relating to both colour and morphological diversity. While certain aspects of reef aesthetics are not considered by this study, it nevertheless provides evidence that coral restoration can support the return of visual appeal, with potentially important implications for cultural heritage and economic opportunities related to tourism. Reef restoration practitioners could target aesthetic value as a crucial aspect of coral ecosystem functioning, with specifically designed restoration interventions. Ultimately, restoring coral reefs that exhibit high aesthetic value will contribute to the maintenance of ecosystem services that are culturally significant and economically valuable for coastal communities worldwide.

## Supplementary Information

Supplementary Information.

## Data Availability

Data and original code supporting the findings in this paper are publicly available on GitHub (https://github.com/alisagita/Aesthetic-value-of-restored-reefs). Any additional information required to reanalyze the data reported in this paper is available from the lead contact upon request.
