# Supplementary material for: Benthic communities on restored coral reefs confer equivalent aesthetic value to healthy reefs
Source: Sci Rep. 2025 Jul 1;15:20790. doi: 10.1038/s41598-025-06373-3 (PMC12215959; doi:10.1038/s41598-025-06373-3)
Supplement: Supplementary file 1 — Supplementary Information. [file 41598_2025_6373_MOESM1_ESM.docx]

**Supplementary information**

**Benthic communities on restored coral reefs confer equivalent aesthetic value to healthy reefs**

Cut Aja Gita Alisa*, Tries B. Razak, Nicolas Mouquet, Nicholas A. J. Graham, Christopher R. Hemingson, David Mouillot, Mars Coral Restoration Project monitoring team, Beginer Subhan, Neviaty P. Zamani, Rindah Talitha Vida, Timothy A.C. Lamont*.

*Correspondance to: [gitaalisa0808@gmail.com](mailto:gitaalisa0808@gmail.com) and [tim.lamont@lancaster.ac.uk](mailto:tim.lamont@lancaster.ac.uk)

Data and original code supporting the findings in this paper are publicly available on GitHub (https://github.com/alisagita/Aesthetic-value-of-restored-reefs). Any additional information required to reanalyse the data reported in this paper is available from the lead contact upon request.

**Table S1.** Site depths and photograph dates.

| **Habitat** | **Site** | **Depth (m)** | **Photograph dates** |
| --- | --- | --- | --- |
| Healthy | Salisi Kecil South | 4 | 31 August 2022 |
|  | Gusung Bontosua Healthy 1 | 4 | 1 September 2022 |
|  | Gusung Bontosua Healthy 2 | 2.5 | 2 September 2022 |
|  | Good Control 3 | 4.3 | 5 September 2022 |
|  | Gusung Bontosua Healthy 3 | 2 | 9 September 2022 |
|  | Good Control 2 | 3.1 | 25 May 2023 |
| Restored | Central West Sector | 2 | 1 September 2022 |
|  | Block 3 | 4 | 5 September 2022 |
|  | Block 4 | 3 | 5 September 2022 |
|  | Block 6 | 3 | 5 September 2022 |
|  | Block 7 | 2 | 7 September 2022 |
|  | Salisi Kecil North | 3 | 8 September 2022 |
| Degraded | South East Sector | 3.4 | 2 September 2022 |
|  | Bad Control 3 | 3.3 | 6 September 2022 |
|  | Salisi Kecil East | 4 | 8 September 2022 |
|  | Gusung Bontosua Degraded | 2.7 | 9 September 2022 |
|  | North Central Sector | 4 | 25 May 2023 |
|  | Central East Sector | 3.4 | 25 May 2023 |


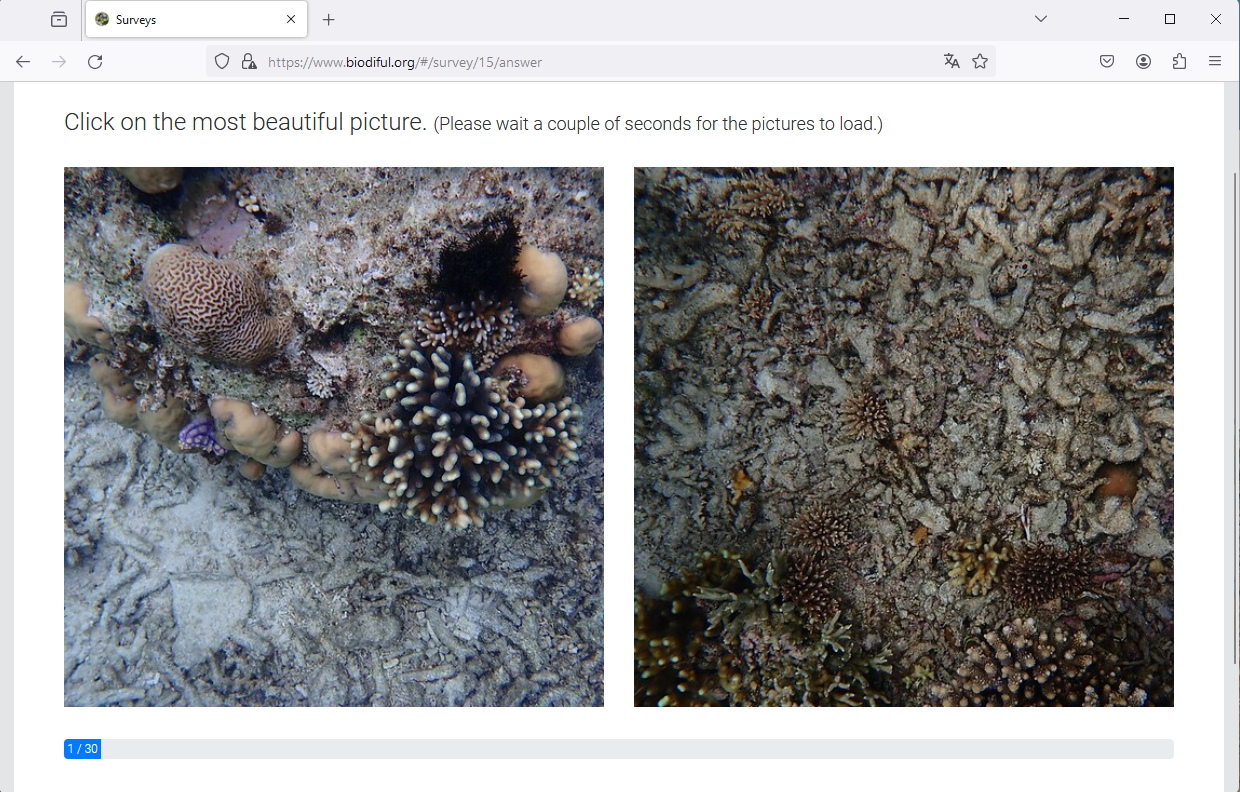


**Fig S1.** Screenshot of the presentation of paired photographs as presented in the online survey on a computer.


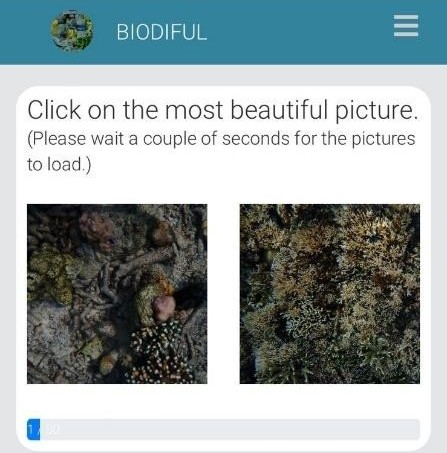


**Fig S2.** Screenshot of the presentation of paired photographs as presented in the online survey on a mobile phone (portrait mode).


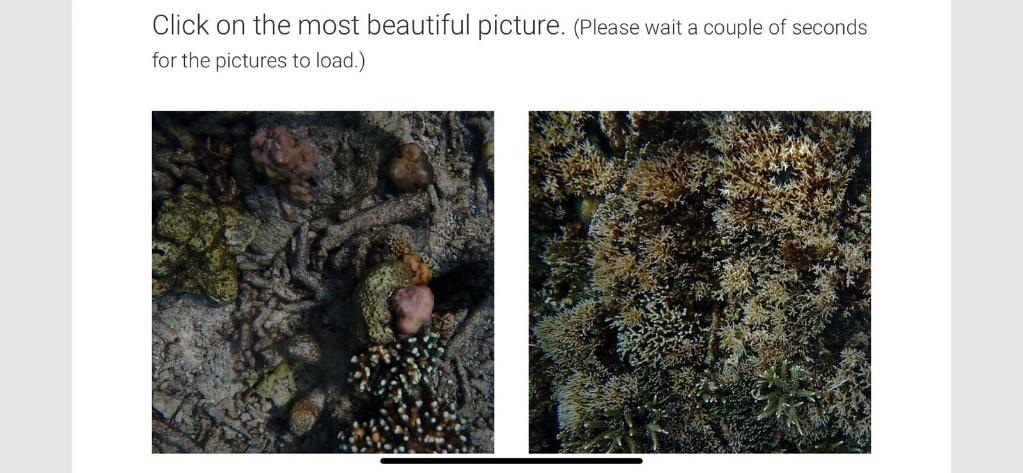


**Fig S3.** Screenshot of the presentation of paired photographs as presented in the online survey on a mobile phone (landscape mode).

**List of questions and possible responses in the online survey**

1. Gender?
   - Female
   - Male
   - Other
2. Age?

1 to 99

1. Country?

(Drop-down possible answers)

1. Highest level of education attained?
   - Professional certificate
   - High school graduate
   - Associate’s and/or Bachelor’s degree
   - Master’s degree
   - Doctoral or Professional degree
2. Have you ever been diving or snorkeling?
   - Only diving
   - Only snorkeling
   - Both diving and snorkeling
   - Never
3. How would you evaluate your knowledge of coral reefs?
   - Poor
   - Low
   - Average
   - Good
   - Excellent
4. Do you have problems with color perception?
   - Yes
   - No

**Table S2.** Non-exhaustive list of example online channels where the survey was shared. All participants were encouraged to share the survey widely amongst their own networks, precluding the possibility of accurately tracking all platforms on which the survey was shared.

| **Date shared** | **Channel** | **Group name** | **Info** |
| --- | --- | --- | --- |
| May 2023 | WhatsApp | Personal groups | Family and friends groups |
| May 2023 | WhatsApp | Common interest group | People interested in coral restoration, several hundred |
| May 2023 | Twitter | General Tweet | Publically visible Tweet |
| May 2023 | Email | University email list | Lab group and contacts |
| May 2023 | WhatsApp | Undergraduate class group | Friends group |
| May 2023 | WhatsApp | Diving club group | Divers group |
| May 2023 | WhatsApp | Personal groups | People interested in coastal ecosystems |
| May 2023 | WhatsApp | University students’ group | Several hundreds post-graduates students |
| May 2023 | WhatsApp | Asking several friends to share | General friends |
| May 2023 | Twitter | General Tweet | Public tweet |
| July 2023 | Linkedin | General Post | Public post |
| July 2023 | Instagram | Instagram Story | Public post by an account with 1,300 followers |
| July 2023 | Email | University staff email list | University department email list |
| July 2023 | Email | University email list | University department email list |
| July 2023 | Email | University email list | University department email list |
| July 2023 | Email | Research Centre email list | University department email list |
| July 2023 | Email | University email list | University department email list |
| July 2023 | Email | Research society email list | Research society email list |
| July 2023 | Email | University email list | University department email list |
| June 2023 | Email | University email list | University department email list |
| June 2023 | Email | Primary school interest group | Primary school students in UK and their families |
| June 2023 | Email | Primary school interest group | Primary and secondary school students around USA and Canada, and their teachers and families |
| May 2023 | Email | University email list | University department email list |
| May 2023 | Instagram | Personal social media post | Public post by an account with 500 followers |
| May 2023 | Strava | Personal social media post | Public post by an account with 400 followers |
| May 2023 | Instagram story | Personal social media post | Public post by an account with 500 followers |
| June 2023 | Instagram story | Personal social media post | Public post by an account with 500 followers |
| June 2023 | Instagram story | Personal social media post | Public post by an account with 500 followers |
| May 2023 | Twitter | General Tweet | Public tweet |
| May 2023 | Email | Focus group netwrok mailing list | Research society email list |
| May 2023 | Web | Focus group netwrok mailing list | Research society email list |
| May 2023 | Email | Focus group netwrok mailing list | Research society email list |
| May 2023 | Email | University lab group email list | University department email list |
| May 2023 | Email | Focus group netwrok mailing list (French) | About 5000 subscibers |
| May 2023 | Email | Focus group netwrok mailing list (English) | About 2000 subscibers |
| May 2023 | WhatsApp | Personal groups | Family and friends |

**Table S3.** Breakdown of the number of participants by each sociocultural variable

| **No** | **Variable** | **Number of participants** |
| --- | --- | --- |
| 1 | **Gender** | |
|  | Female | 2063 |
|  | Male | 1237 |
|  | Other | 48 |
| **2** | **Age class** | |
|  | 0-12 | 42 |
|  | 13-18 | 84 |
|  | 19-30 | 1542 |
|  | 31-59 | 1445 |
|  | 60-100 | 235 |
| **3** | **Country** |  |
|  | France | 954 |
|  | Indonesia | 541 |
|  | UK | 468 |
|  | USA | 330 |
|  | Australia | 239 |
|  | Italy | 91 |
|  | Canada | 85 |
|  | Germany | 58 |
|  | French Southern Territories | 56 |
|  | Réunion | 52 |
|  | Netherlands | 37 |
|  | Switzerland | 35 |
|  | Spain | 26 |
|  | Belgium | 22 |
|  | Philippines | 20 |
|  | Other 92 countries | 334 |
| **4** | **Highest level of education** | |
|  | Secondary | 161 |
|  | High school | 348 |
|  | Bachelor | 928 |
|  | Master | 1083 |
|  | PhD | 828 |
| **5** | **Experience of diving** |  |
|  | Diving | 1785 |
|  | Only Snorkeling | 816 |
|  | None | 747 |
| **6** | **Knowledge of corals** |  |
|  | Poor | 424 |
|  | Low | 839 |
|  | Average | 880 |
|  | Good | 776 |
|  | Excellent | 429 |
| **7** | **Colour blind** |  |
|  | Yes | 96 |
|  | No | 3252 |

**Fig. S4** The distribution of Elo scores for the 300 evaluated photographs (in red) and the 883 predicted photographs (in blue). **
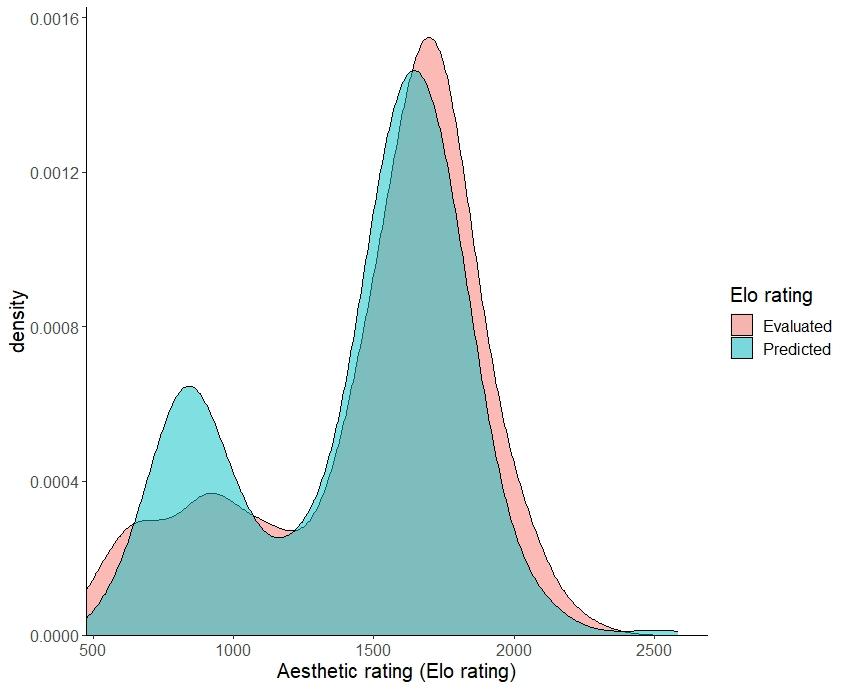
**

**Fig. S5.** Summary of the socio-cultural background of the 3,348 participants. Shown are, panel (a) gender, (b) colour blindness, (c) age, (d) age class, (e) highest level of education, (f) experience of diving, (g) knowledge of corals, and (h) country.

**
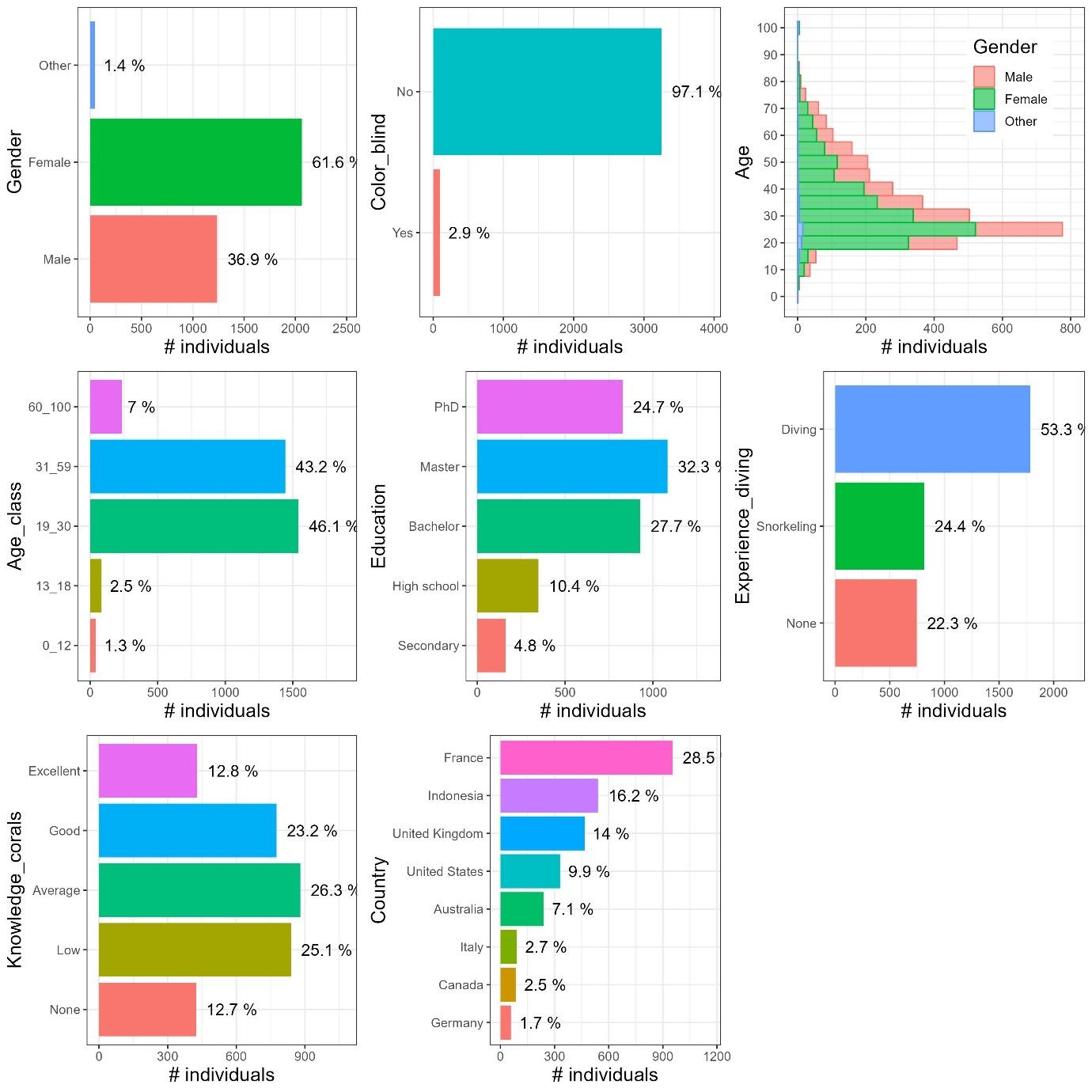
**

**Table S4-a.** Outputs from generalised linear mixed-effects investigating the impact of socio-cultural variables on aesthetic rating.

| **Variables** | **Chisq** | **Df** | **Pr..Chisq.** | **Significance** |
| --- | --- | --- | --- | --- |
| Gender | 0.487 | 2 | 0.784 | ns. |
| Age_class | 0.363 | 4 | 0.985 | ns. |
| Education | 2.506 | 4 | 0.644 | ns. |
| Experience_diving | 1.502 | 2 | 0.472 | ns. |
| Knowledge_corals | 2.516 | 4 | 0.642 | ns. |
| Country | 0.622 | 5 | 0.987 | ns. |

**Table S4-b.** Outputs from generalised linear mixed-effects investigating two-way interactions between each socio-cultural variables on aesthetic rating.

| **Variables** | **Chisq** | **Df** | **Pr..Chisq** | **Interaction** | **Significance** |
| --- | --- | --- | --- | --- | --- |
| Gender | 0.448 | 2 | 0.799 | Gender * Age_class | ns. |
| Age_class | 1.628 | 4 | 0.804 | Gender * Age_class | ns. |
| Gender:Age_class | 3.739 | 8 | 0.880 | Gender * Age_class | ns. |
| Gender | 0.492 | 2 | 0.782 | Gender * Education | ns. |
| Education | 3.555 | 4 | 0.470 | Gender * Education | ns. |
| Gender:Education | 1.563 | 8 | 0.992 | Gender * Education | ns. |
| Gender | 0.413 | 2 | 0.814 | Gender * Experience_diving | ns. |
| Experience_diving | 1.026 | 2 | 0.599 | Gender * Experience_diving | ns. |
| Gender:Experience_diving | 0.428 | 4 | 0.980 | Gender * Experience_diving | ns. |
| Gender | 0.400 | 2 | 0.819 | Gender * Knowledge_corals | ns. |
| Knowledge_corals | 2.331 | 4 | 0.675 | Gender * Knowledge_corals | ns. |
| Gender:Knowledge_corals | 1.323 | 8 | 0.995 | Gender * Knowledge_corals | ns. |
| Gender | 0.393 | 2 | 0.822 | Gender * Country | ns. |
| Country | 1.030 | 5 | 0.960 | Gender * Country | ns. |
| Gender:Country | 2.010 | 9 | 0.991 | Gender * Country | ns. |
| Age_class | 0.668 | 4 | 0.955 | Age_class * Education | ns. |
| Education | 2.540 | 4 | 0.637 | Age_class * Education | ns. |
| Age_class:Education | 1.867 | 13 | 1.000 | Age_class * Education | ns. |
| Age_class | 1.647 | 4 | 0.800 | Age_class * Experience_diving | ns. |
| Experience_diving | 1.065 | 2 | 0.587 | Age_class * Experience_diving | ns. |
| Age_class:Experience_diving | 1.524 | 8 | 0.992 | Age_class * Experience_diving | ns. |
| Age_class | 1.421 | 4 | 0.841 | Age_class * Knowledge_corals | ns. |
| Knowledge_corals | 2.144 | 4 | 0.709 | Age_class * Knowledge_corals | ns. |
| Age_class:Knowledge_corals | 3.487 | 15 | 0.999 | Age_class * Knowledge_corals | ns. |
| Age_class | 1.000 | 4 | 0.910 | Age_class * Country | ns. |
| Country | 0.404 | 5 | 0.995 | Age_class * Country | ns. |
| Age_class:Country | 4.952 | 20 | 1.000 | Age_class * Country | ns. |
| Education | 3.409 | 4 | 0.492 | Education * Experience_diving | ns. |
| Experience_diving | 0.970 | 2 | 0.616 | Education * Experience_diving | ns. |
| Education:Experience_diving | 3.669 | 8 | 0.886 | Education * Experience_diving | ns. |
| Education | 3.148 | 4 | 0.533 | Education * Knowledge_corals | ns. |
| Knowledge_corals | 2.028 | 4 | 0.731 | Education * Knowledge_corals | ns. |
| Education:Knowledge_corals | 4.002 | 16 | 0.999 | Education * Knowledge_corals | ns. |
| Education | 3.055 | 4 | 0.549 | Education * Country | ns. |
| Country | 0.609 | 5 | 0.988 | Education * Country | ns. |
| Education:Country | 8.318 | 20 | 0.990 | Education * Country | ns. |
| Experience_diving | 1.397 | 2 | 0.497 | Experience_diving * Knowledge_corals | ns. |
| Knowledge_corals | 2.711 | 4 | 0.607 | Experience_diving * Knowledge_corals | ns. |
| Experience_diving:Knowledge_corals | 3.989 | 8 | 0.858 | Experience_diving * Knowledge_corals | ns. |
| Experience_diving | 0.987 | 2 | 0.611 | Experience_diving * Country | ns. |
| Country | 1.003 | 5 | 0.962 | Experience_diving * Country | ns. |
| Experience_diving:Country | 3.334 | 10 | 0.972 | Experience_diving * Country | ns. |
| Knowledge_corals | 2.436 | 4 | 0.656 | Knowledge_corals * Country | ns. |
| Country | 1.140 | 5 | 0.950 | Knowledge_corals * Country | ns. |
| Knowledge_corals:Country | 4.338 | 20 | 1.000 | Knowledge_corals * Country | ns. |

**Table S5.** Thumbprint of 64 colour bins created by the R package *colordistance* and the mean ± standard deviation proportion of each bin represented in each habitat. The ‘Bin colour code in R,G,B’ below was obtained by converting hex codes of colours generated from the *getHistColors* command in the package *colordistance*.

| **Bin** | **Bin colour code in R,G,B** | **Healthy** | **Restored** | **Degraded** |
| --- | --- | --- | --- | --- |
| 1 | 32, 32, 32 | 45.815 ± 11.966 | 49.477 ± 12.823 | 37.932 ± 13.718 |
| 2 | 96, 32, 32 | 2.754 ± 1.474 | 3.888 ± 2.730 | 3.092 ± 1.935 |
| 3 | 159, 32, 32 | 0.002 ± 0.008 | 0.004 ± 0.053 | < 0.001 ± < 0.001 |
| 4 | 223, 32, 32 | 0.000 ± 0.000 | < 0.001 ± < 0.001 | 0.000 ± 0.000 |
| 5 | 32, 96, 32 | 0.514 ± 0.420 | 0.469 ± 0.475 | 0.000 ± 0.000 |
| 6 | 96, 96, 32 | 3.627 ± 1.955 | 5.132 ± 3.712 | 3.715 ± 2.462 |
| 7 | 159, 96, 32 | 0.108 ± 0.179 | 0.207 ± 0.524 | 0.049 ± 0.190 |
| 8 | 223, 96, 32 | < 0.001 ± 0.001 | < 0.001 ± < 0.001 | < 0.001 ± < 0.001 |
| 9 | 32, 159, 32 | 0.000 ± 0.000 | 0.000 ± 0.000 | 0.000 ± 0.000 |
| 10 | 96, 159, 32 | 0.001 ± 0.003 | 0.006 ± 0.028 | < 0.001 ± 0.002 |
| 11 | 159, 159, 32 | 0.002 ± 0.006 | 0.007 ± 0.027 | 0.001 ± 0.003 |
| 12 | 223, 159, 32 | < 0.001 ± 0.004 | < 0.001 ± 0.001 | < 0.001 ± < 0.001 |
| 13 | 32, 223, 32 | 0.000 ± 0.000 | 0.000 ± 0.000 | 0.000 ± 0.000 |
| 14 | 96, 223, 32 | 0.000 ± 0.000 | 0.000 ± 0.000 | 0.000 ± 0.000 |
| 15 | 159, 223, 32 | < 0.001 ± < 0.001 | 0.000 ± 0.000 | 0.000 ± 0.000 |
| 16 | 223, 223, 32 | < 0.001 ± < 0.001 | 0.000 ± 0.000 | 0.000 ± 0.000 |
| 17 | 32, 32, 96 | 3.630 ± 2.292 | 3.549 ± 4.103 | 2.465 ± 2.186 |
| 18 | 255, 255, 255 | 0.341 ± 0.271 | 0.253 ± 0.261 | 0.265 ± 0.233 |
| 19 | 159, 32, 96 | < 0.001 ± 0.001 | 0.001 ± 0.023 | < 0.001 ± < 0.001 |
| 20 | 223, 32, 96 | 0.000 ± 0.000 | < 0.001 ± < 0.001 | 0.000 ± 0.000 |
| 21 | 32, 96, 96 | 1.978 ± 1.438 | 1.589 ± 1.495 | 1.707 ± 1.530 |
| 22 | 96, 96, 96 | 19.200 ± 4.746 | 16.025 ± 5.887 | 35.477 ± 10.564 |
| 23 | 159, 96, 96 | 2.809 ± 1.820 | 2.717 ± 1.609 | 1.715 ± 1.623 |
| 24 | 223, 96, 96 | 0.002 ± 0.004 | 0.002 ± 0.026 | < 0.001 ± 0.001 |
| 25 | 32, 159, 96 | < 0.001 ± 0.001 | < 0.001 ± < 0.001 | < 0.001 ± < 0.001 |
| 26 | 96, 159, 96 | 0.544 ± 0.350 | 0.509 ± 0.446 | 0.287 ± 0.274 |
| 27 | 159, 159, 96 | 3.940 ± 2.311 | 3.586 ± 1.807 | 1.752 ± 1.535 |
| 28 | 223, 159, 96 | 0.086 ± 0.149 | 0.075 ± 0.148 | 0.011 ± 0.067 |
| 29 | 32, 223, 96 | 0.000 ± 0.000 | 0.000 ± 0.000 | 0.000 ± 0.000 |
| 30 | 96, 223, 96 | 0.000 ± 0.000 | 0.000 ± 0.000 | < 0.001 ± < 0.001 |
| 31 | 159, 223, 96 | 0.001 ± 0.003 | 0.002 ± 0.012 | < 0.001 ± < 0.001 |
| 32 | 223, 223, 96 | 0.002 ± 0.008 | 0.002 ± 0.010 | < 0.001 ± 0.001 |
| 33 | 32, 32, 159 | 0.013 ± 0.038 | 0.014 ± 0.059 | 0.001 ± 0.002 |
| 34 | 96, 32, 159 | < 0.001 ± < 0.001 | < 0.001 ± 0.001 | < 0.001 ± < 0.001 |
| 35 | 159, 32, 159 | 0.000 ± 0.000 | 0.000 ± 0.000 | 0.000 ± 0.000 |
| 36 | 223, 32, 159 | 0.000 ± 0.000 | 0.000 ± 0.000 | 0.000 ± 0.000 |
| 37 | 32, 96, 159 | 0.074 ± 0.114 | 0.093 ± 0.239 | 0.009 ± 0.015 |
| 38 | 96, 96, 159 | 1.088 ± 1.087 | 0.967 ± 1.136 | 1.98 ± 3.766 |
| 39 | 159, 96, 159 | 0.127 ± 0.181 | 0.096 ± 0.194 | 0.099 ± 0.251 |
| 40 | 223, 96, 159 | < 0.001 ± < 0.001 | < 0.001 ± 0.002 | < 0.001 ± < 0.001 |
| 41 | 32, 159, 159 | 0.002 ± 0.004 | 0.002 ± 0.003 | < 0.001 ± < 0.001 |
| 42 | 96, 159, 159 | 1.021 ± 0.826 | 0.952 ± 1.004 | 1.418 ± 2.460 |
| 43 | 159, 159, 159 | 7.960 ± 4.329 | 6.739 ± 4.437 | 7.034 ± 6.186 |
| 44 | 223, 159, 159 | 0.870 ± 0.970 | 0.581 ± 0.678 | 0.136 ± 0.356 |
| 45 | 32, 223, 159 | 0.000 ± 0.000 | 0.000 ± 0.000 | 0.000 ± 0.000 |
| 46 | 96, 223, 159 | < 0.001 ± 0.001 | < 0.001 ± 0.001 | < 0.001 ± < 0.001 |
| 47 | 159, 223, 159 | 0.203 ± 0.199 | 0.222 ± 0.228 | 0.024 ± 0.077 |
| 48 | 223, 223, 159 | 1.073 ± 1.268 | 0.727 ± 0.778 | 0.108 ± 0.491 |
| 49 | 32, 32, 223 | < 0.001 ± < 0.001 | < 0.001 ± < 0.001 | < 0.001 ± < 0.001 |
| 50 | 96, 32, 223 | < 0.001 ± < 0.001 | 0.000 ± 0.000 | 0.000 ± 0.000 |
| 51 | 159, 32, 223 | 0.000 ± 0.000 | 0.000 ± 0.000 | 0.000 ± 0.000 |
| 52 | 223, 32, 223 | 0.000 ± 0.000 | 0.000 ± 0.000 | 0.000 ± 0.000 |
| 53 | 32, 96, 223 | 0.001 ± 0.004 | 0.001 ± 0.005 | < 0.001 ± < 0.001 |
| 54 | 96, 96, 223 | 0.004 ± 0.014 | 0.003 ± 0.013 | < 0.001 ± < 0.001 |
| 55 | 159, 96, 223 | < 0.001 ± < 0.001 | < 0.001 ± 0.001 | 0.000 ± 0.000 |
| 56 | 223, 96, 223 | < 0.001 ± < 0.001 | 0.000 ± 0.000 | 0.000 ± 0.000 |
| 57 | 32, 159, 223 | 0.001 ± 0.002 | 0.001 ± 0.004 | < 0.001 ± < 0.001 |
| 58 | 96, 159, 223 | 0.038 ± 0.054 | 0.040 ± 0.097 | 0.003 ± 0.009 |
| 59 | 159, 159, 223 | 0.280 ± 0.275 | 0.331 ± 0.475 | 0.172 ± 0.446 |
| 60 | 223, 159, 223 | 0.021 ± 0.030 | 0.013 ± 0.028 | 0.003 ± 0.011 |
| 61 | 32, 223, 223 | < 0.001 ± < 0.001 | < 0.001 ± < 0.001 | 0.000 ± 0.000 |
| 62 | 96, 223, 223 | 0.002 ± 0.003 | 0.002 ± 0.004 | < 0.001 ± < 0.001 |
| 63 | 159, 223, 223 | 0.263 ± 0.228 | 0.385 ± 0.554 | 0.049 ± 0.117 |
| 64 | 223, 223, 223 | 1.604 ± 1.817 | 1.326 ± 1.746 | 0.148 ± 0.440 |

**Table S6.**

Colour groupings, formed from combinations of 64 bins created by the R package *colordistance* and the mean ± standard deviation proportion of each colour group represented in each habitat. The ‘Bin colour code in R,G,B’ below below was obtained by converting hex codes of colours generated from the *getHistColors* command in package *colordistance*.

| **Colour** | **Bins** | **Bin colour code in R,G,B** | **Healthy** | **Restored** | **Degraded** |
| --- | --- | --- | --- | --- | --- |
| **Black** | 1 | 32, 32, 32 | 45.815 ± 11.966 | 49.477 ± 12.823 | 37.932 ± 13.718 |
| **Total Black** | | | **45.815 ± 11.966** | **49.477 ± 12.823** | **37.932 ± 13.718** |
| **Grey** | 22 | 96, 96, 96 | 19.200 ± 4.746 | 16.025 ± 5.887 | 35.477 ± 10.564 |
|  | 43 | 159, 159, 159 | 7.960 ± 4.329 | 6.739 ± 4.437 | 7.034 ± 6.186 |
|  | 64 | 223, 223, 223 | 1.604 ± 1.817 | 1.326 ± 1.746 | 0.148 ± 0.44 |
| **Total grey** | | | **28.764 ± 10.892** | **24.09 ± 12.053** | **42.659 ± 19.976** |
| **Blue** | 17 | 32, 32, 96 | 3.630 ± 2.292 | 3.549 ± 4.103 | 2.465 ± 2.186 |
|  | 33 | 32, 32, 159 | 0.013 ± 0.038 | 0.014 ± 0.059 | 0.001 ± 0.002 |
|  | 37 | 32, 96, 159 | 0.074 ± 0.114 | 0.093 ± 0.239 | 0.009 ± 0.015 |
|  | 41 | 32, 159, 159 | 0.002 ± 0.004 | 0.002 ± 0.003 | 0.000 ± 0.000 |
|  | 53 | 32, 96, 223 | 0.001 ± 0.004 | 0.001 ± 0.005 | 0.000 ± 0.000 |
|  | 57 | 32, 159, 223 | 0.001 ± 0.002 | 0.001 ± 0.004 | 0.000 ± 0.000 |
|  | 58 | 96, 159, 223 | 0.038 ± 0.054 | 0.04 ± 0.097 | 0.003 ± 0.009 |
|  | 61 | 32, 223, 223 | 0.000 ± 0.000 | 0.000 ± 0.000 | 0.000 ± 0.000 |
|  | 62 | 96, 223, 223 | 0.002 ± 0.003 | 0.002 ± 0.004 | 0.000 ± 0.000 |
| **Total blue** | | | **3.761 ± 2.511** | **3.702 ± 4.514** | **2.478 ± 2.212** |
| **Yellow** | 2 | 96, 32, 32 | 2.754 ± 1.474 | 3.888 ± 2.730 | 3.092 ± 1.935 |
|  | 3 | 159, 32, 32 | 0.002 ± 0.008 | 0.004 ± 0.053 | 0.000 ± 0.000 |
|  | 4 | 223, 32, 32 | 0.000 ± 0.000 | 0.000 ± 0.000 | 0.000 ± 0.000 |
|  | 7 | 159, 96, 32 | 0.108 ± 0.179 | 0.207 ± 0.524 | 0.049 ± 0.190 |
|  | 8 | 223, 96, 32 | 0.000 ± 0.000 | 0.000 ± 0.000 | 0.000 ± 0.000 |
| **Total yellow** | | | **1.864 ± 1.661** | **4.099 ± 3.307** | **3.141 ± 2.125** |
| **Green** | 5 | 32, 96, 32 | 0.514 ± 0.420 | 0.469 ± 0.475 | 0.000 ± 0.000 |
|  | 6 | 96, 96, 32 | 3.627 ± 1.955 | 5.132 ± 3.712 | 3.715 ± 2.462 |
|  | 9 | 32, 159, 32 | 0.000 ± 0.000 | 0.000 ± 0.000 | 0.000 ± 0.000 |
|  | 10 | 96, 159, 32 | 0.001 ± 0.003 | 0.006 ± 0.028 | 0.000 ± 0.000 |
|  | 13 | 32, 223, 32 | 0.000 ± 0.000 | 0.000 ± 0.000 | 0.000 ± 0.000 |
|  | 14 | 96, 223, 32 | 0.000 ± 0.000 | 0.000 ± 0.000 | 0.000 ± 0.000 |
|  | 15 | 159, 223, 32 | 0.000 ± 0.000 | 0.000 ± 0.000 | 0.000 ± 0.000 |
|  | 25 | 32, 159, 96 | 0.000 ± 0.000 | 0.000 ± 0.000 | 0.000 ± 0.000 |
|  | 26 | 96, 159, 96 | 0.544 ± 0.350 | 0.509 ± 0.446 | 0.287 ± 0.274 |
|  | 29 | 32, 223, 96 | 0.000 ± 0.000 | 0.000 ± 0.000 | 0.000 ± 0.000 |
| **Total green** | | | **4.686 ± 2.728** | **6.116 ± 4.661** | **4.002 ± 2.736** |

**Backward stepwise analysis**

**Table S7-a.** First iteration of backward stepwise correlation matrix (Pearson correlation) among all features. **‘**Simpson’ and ‘Grey’ were correlated exceeding the threshold (-0.7), so one of these features needed to be removed.

|  | Grey | Simpson | Black | Blue | Green | Yellow | Number of colour | Number of morpho-logies | % Live coral |
| --- | --- | --- | --- | --- | --- | --- | --- | --- | --- |
| Grey | 1 | -0.91 | 0.68 | 0.64 | -0.08 | 0.53 | 0.44 | 0.01 | 0.53 |
| Simpson | -0.91 | 1 | -0.85 | -0.51 | 0.11 | -0.36 | -0.46 | 0.03 | -0.55 |
| Black | 0.68 | -0.85 | 1 | 0.39 | -0.31 | 0.23 | 0.28 | -0.29 | 0.34 |
| Blue | 0.64 | -0.51 | 0.39 | 1 | -0.55 | 0.69 | 0.14 | -0.28 | 0.16 |
| Green | -0.08 | 0.11 | -0.31 | -0.55 | 1 | -0.5 | 0.11 | 0.46 | 0.11 |
| Yellow | 0.53 | -0.36 | 0.23 | 0.69 | -0.5 | 1 | 0.03 | -0.28 | 0.03 |
| Number of colour | 0.44 | -0.46 | 0.28 | 0.14 | 0.11 | 0.03 | 1 | 0.34 | 0.62 |
| Number of morphologies | 0.01 | 0.03 | -0.29 | -0.28 | 0.46 | -0.28 | 0.34 | 1 | 0.41 |
| % Live coral | 0.53 | -0.55 | 0.34 | 0.16 | 0.11 | 0.03 | 0.62 | 0.41 | 1 |

**Table S7-b.** Individually tested ‘Simpson’ and ‘Grey’ correlations with predicted aesthetic rating. We kept ‘Simpson’ due to its higher correlation with predicted aesthetic rating than ‘Grey’, and ‘Grey’ was eliminated for next iteration.

|  | Predicted aesthetic rating |
| --- | --- |
| Simpson | 0.73 |
| Grey | -0.71 |

**Table S7-c.** Second iteration of backward stepwise correlation matrix (Pearson correlation) after ‘Grey’ was eliminated. There were no more features that correlated exceeding the threshold (0.7), so no more features were removed.

|  | Simpson | Black | Blue | Green | Yellow | Number of colour | Number of morpho-logies | % Live coral |
| --- | --- | --- | --- | --- | --- | --- | --- | --- |
| Simpson | 1 | 0.68 | 0.64 | -0.08 | 0.53 | 0.44 | 0.01 | 0.53 |
| Black | 0.68 | 1 | 0.39 | -0.31 | 0.23 | 0.28 | -0.29 | 0.34 |
| Blue | 0.64 | 0.39 | 1 | -0.55 | 0.69 | 0.14 | -0.28 | 0.16 |
| Green | -0.08 | -0.31 | -0.55 | 1 | -0.5 | 0.11 | 0.46 | 0.11 |
| Yellow | 0.53 | 0.23 | 0.69 | -0.5 | 1 | 0.03 | -0.28 | 0.03 |
| Number of colour | 0.44 | 0.28 | 0.14 | 0.11 | 0.03 | 1 | 0.34 | 0.62 |
| Number of morphologies | 0.01 | -0.29 | -0.28 | 0.46 | -0.28 | 0.34 | 1 | 0.41 |
| % Live coral | 0.53 | 0.34 | 0.16 | 0.11 | 0.03 | 0.62 | 0.41 | 1 |

**Table S7-d.**

Backward stepwise linear regression model: Non-significant features (p > 0.05) were eliminated in each iteration. Yellow was eliminated in the first step, green in the second step, leaving six significant features remaining.

|  | 1^st^ model | 2^nd^ model | 3^rd^ model |
| --- | --- | --- | --- |
|  | *p* | *p* | *p* |
| Simpson | < 0.05 | < 0.05 | < 0.05 |
| Black | < 0.05 | < 0.05 | < 0.05 |
| Blue | < 0.05 | < 0.05 | < 0.05 |
| Green (eliminated in first step) | 0.22 |  |  |
| Yellow (eliminated in second step) | < 0.05 | 0.06 |  |
| Number of colour | < 0.05 | < 0.05 | < 0.05 |
| Number of morphologies | < 0.05 | < 0.05 | < 0.05 |
| % Live coral | < 0.05 | < 0.05 | < 0.05 |

**Table S8.** Outputs from generalised linear mixed-effects and linear mixed-effects models investigating the impact of habitat type on predicted aesthetic rating and six significant image features. Untransformed model estimates and standard errors are provided for the fixed effect (habitat type); variances ± standard deviations are provided for random terms. Significant models and post-hoc comparisons (p≤0.05) are displayed in bold.

| Fixed effect (habitat type) | Model estimates | | Post-hoc comparison | Tukey’s HSD *p* |
| --- | --- | --- | --- | --- |
| Aesthetic rating (Elo score)  (Linear Mixed Model: χ2 = 43.85, df = 2, p < 0.001) | | | | |
| Healthy | 1638.86 ± 43.25 | | Healthy vs restored | 0.806 |
| Restored | 1677.11 ± 43.20 | | **Healthy vs degraded** | **< 0.001** |
| Degraded | 997.44 ± 43.16 | | **Restored vs degraded** | **< 0.001** |
| Random effects: Site = 10214 ± 101.1 | | | | |
| Simpson diversity of colours  (Gamma-distributed Generalised Linear Mixed Model χ2 = 41.508, df = 3, p < 0.001) | | | | |
| Healthy | -0.800 ± 0.087 | | Healthy vs restored | 0.5828 |
| Restored | -0.678 ± 0.088 | | **Healthy vs degraded** | **0.0217** |
| Degraded | -1.129 ± 0.088 | | **Restored vs degraded** | **< 0.001** |
| Random effects: Site = 0.0053 ± 0.073 | | | | |
| Number of colour categories present  (Poisson-distributed Generalised Linear Mixed Model χ2 = 95.169 , df = 3, p < 0.001) | | | | |
| Healthy | 2.348 ± 0.064 | | Healthy vs restored | 0.579 |
| Restored | 2.257 ± 0.064 | | **Healthy vs degraded** | **0.007** |
| Degraded | 2.072 ± 0.065 | | Restored vs degraded | 0.108 |
| Random effects: Site = 0.0229 ± 0.15 | | | | |
| Percent of image in blue colour category  (Gamma-distributed Generalised Linear Mixed Model χ2 = 8.649 , df = 3, p = 0.035) | | | | |
| Healthy | 1.301 ± 0.467 | | Healthy vs restored | 0.383 |
| Restored | 0.427 ± 0.467 | | Healthy vs degraded | 0.674 |
| Degraded | 0.741 ± 0.467 | | Restored vs degraded | 0.883 |
| Random effect: Site = 0.524 ± 0.724 | | | | |
| Percent live hard coral cover  (Linear Mixed Model: χ2 = 46.39, df = 2, p < 0.001) | | | | |
| Healthy | | 70.430 ± 3.311 | Healthy vs restored | 0.116 |
| Restored | | 61.142 ± 3.306 | **Healthy vs degraded** | **< 0.001** |
| Degraded | | 11.707 ± 3.301 | **Restored vs degraded** | **< 0.001** |
| Random effect: Site = 56.56 ± 7.521 | | | | |

| Percent of image in shadow (proxy for structural complexity)  (Linear Mixed Model χ2 = 3.265 , df = 2 , p = 0.195) | | | |
| --- | --- | --- | --- |
| Healthy | 45.628 ± 4.899 | LMM not significant | |
| Restored | 49.714 ± 4.899 |  |  |
| Degraded | 37.932 ± 4.898 |  |  |
| Random effect: Site = 143.1 ± 11.961 | | | |
| Number of coral morphologies present  (Poisson-distributed Generalised Linear Mixed Model χ2 = 18.033, df = 3, p < 0.001) | | | |
| Healthy | 0.970 ± 0.247 | Healthy vs restored | 0.928 |
| Restored | 0.841 ± 0.248 | **Healthy vs degraded** | **< 0.001** |
| Degraded | -0.711 ± 0.269 | **Restored vs degraded** | **< 0.001** |
| Random effect: Site = 0.3591 ± 0.5993 | | | |
